# Integrated care for diabetes—a Singapore approach

**DOI:** 10.5334/ijic.810

**Published:** 2012-03-06

**Authors:** Su Qian Yeo, Matthew Harris, F Azeem Majeed

**Affiliations:** Master of Public Health Student, Imperial College London, Reynolds Building, St Dunstan’s Road, London W6 8RP, UK; Academic Clinical Lecturer in Public Health, Imperial College London, Public Health Specialist Registrar, National Health Service, Reynolds Building, St Dunstan’s Road, London W6 8RP, UK; Professor of Primary Care and Head of the Department of Primary Care and Public Health, Imperial College London, Reynolds Building, St Dunstan’s Road, London W6 8RP, UK

**Keywords:** integrated care, integration, diabetes management

## Abstract

**Background:**

The prevalence of diabetes mellitus is 12.7% in Singapore. Managing people with diabetes in the community may be needed to reduce unnecessary utilisation of expensive specialist resources and to reduce hospital waiting times for patients with complications.

**Care practice:**

The Singapore General Hospital (SGH) Delivering on Target (DOT) Programme was launched in 2005 to right-site clinically stable diabetic patients from the hospital to private DOT GPs. The Chronic Disease Management Office (CDMO) was established and a fully customised DOT information technology (IT) system was developed. Three initiatives were implemented: (i) Subsidised drug delivery programme, (ii) Diagnostic tests incentive programme, and (iii) Allied healthcare incentive programme.

**Discussion:**

Right-siting was enabled through patient incentives that eased the burden of out-of-pocket expenditure. Right Siting Officers (RSOs) maintained a general oversight of the patient pathway. The integrated system supported shared care follow-up by enabling DOT GPs to share updates on the patients’ health status with the referring specialists.

**Conclusion:**

A coherent process across all healthcare providers similar to the SGH DOT Programme may facilitate efforts to shift the care for people with diabetes to the community and to provide integrated care. Successful integration may require incentives for institutional partners and patients.

## Introduction

The number of individuals with diabetes worldwide has increased from about 153 million in 1980 to about 347 million in 2008 [1], due in part to population growth, ageing, urbanisation, and rising prevalence of obesity and physical inactivity [2]. This alarming rise in prevalence causes considerable burden on healthcare systems and national economies [3]. Diabetes is associated with many complications, such as retinopathy, nephropathy and lower limb amputation and led to a loss of 20 million disability-adjusted life years worldwide in 2001 alone [4, p. 592]. This has made new models of diabetes care a priority for many health systems.

In most countries, health care systems adhere to the traditional model comprising three organisational levels: primary, secondary and tertiary care. This traditional model may be unsatisfactory to meet the complex and long-term needs of people with diabetes, because they often see many different healthcare professionals across different stages of care [5, p. 64]. In order to minimise the economic burden on health systems and to improve the patient journey, integrated care has been seen by many as a potential solution, facilitating the professional response to the complex needs of people with chronic diseases [6, 7]. Integrated care is implemented differently in different contexts. This paper discusses integrated care for diabetes in Singapore and provides a case study to illustrate the implementation and lessons learned from integrating diabetes care that are transferable to other developed countries.

## Integrated care for diabetes

There are many definitions of integrated care, and many synonyms are used, such as disease management, case management, care management, managed care, transmural care, patient-centred care, and coordinated care [6, 8, 9]. In this paper, integrated care will be defined as “*an organisational process of coordination which seeks to achieve seamless and continuous care, tailored to the patients’ needs and based on a holistic view of the patient *” [10, p. 227].

Over the last seven years, there have been several key policy stages in Singapore’s move towards more integrated care for patients with diabetes. In 2004, the concept of ‘right-siting’ was first used to describe the principle that chronic disease patients should be managed in primary care instead of specialist settings [11]. Right-siting is defined as “patients treated in the most appropriate location by medically competent teams at the lowest possible cost” [12, 13]. In 2005, the National Healthcare Group developed the Chronic Disease Management System (CDMS), a region-wide disease register that synchronises clinical health records—the diabetes mellitus section was completed in 2007 [14, 15]. Also in 2005, the Singapore Health Services (SingHealth) regional cluster initiated the DOT programme, leading to positive health outcomes in diabetic patients [16]. The aim of the DOT Programme was to right-site chronic patients to primary care, and is benchmarked against evidence-based best practice clinical guidelines [17]. Together with the NHG regional cluster, a National DOT Programme was launched in 2008. In July 2011, the National University Hospital (NUH) and Frontier Healthcare, an eight-clinic private GP chain, announced a collaboration in which stable diabetes patients are referred to Frontier clinics by NUH doctors for co-management over two years [18, 19]. The National Electronic Health Record (NEHR) system was launched in 2011 to integrate service delivery and to improve chronic disease management. It may result in cost savings by preventing unnecessary tests and reducing medication errors [20]. The next phase of the NEHR system involves community hospitals, GPs and nursing homes, while private hospitals may be included in the future [21].

## Singapore

Singapore is a small, high-income, multi-ethnic country with a population of about 5 million [22]. Male and female life expectancies are 79 years and 84 years, respectively [23], and it is 6th in the World Health Organization ranking for overall health system performance out of 191 countries [24, 25]. In Singapore, the estimated prevalence of diabetes in adults aged 20–79 years was 12.7% in 2010 [26]. The estimated health expenditure for diabetes in 2010 was US$493 million (£312 million), which was 15% of the total health expenditure [26]. Like many developed countries, such as the USA, the prevalence of diabetes has been rising in Singapore.

## Healthcare system of Singapore

**Figure 1:**
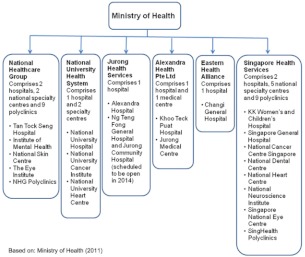
Regional healthcare clusters in Singapore.

The Singapore healthcare system is based on six regional healthcare clusters, as shown in Figure 1. The first two clusters established in 2000 were the National Healthcare Group and SingHealth with the aim of building vertical and horizontal integration between primary care and hospitals [27].

The government funds about a quarter of the total health care costs using taxes, while individuals and their employers pay the rest [28]. Singaporeans have to co-pay for each treatment, medical service, and prescription drug they receive [24].

About 2000 private GPs provide 80% of primary healthcare services, while public outpatient polyclinics run by the National Healthcare Group and SingHealth provide 20%. In contrast, public hospitals provide 80% of hospital care and private hospitals provide 20% [24]. Patients can only receive subsidised inpatient and outpatient care if public polyclinics or the emergency department of public hospitals have first referred them. Self-referral or referral by private GPs to see specific specialists is possible, but no subsidies are provided for medical expenses.

## SGH DOT Programme

In this article, we draw specifically on the SingHealth regional cluster to describe integrated care for diabetes in Singapore as it has the longest experience of this approach and was the forerunner for more universal integrative elements now rolled out across the country. SGH is the largest acute tertiary hospital in Singapore and part of the SingHealth. Before 2005, patients discharged from the SGH Diabetes Centre would be referred to polyclinics, unless they were initially referred by a private GP. Weak links between SGH Endocrinologists and private GPs led to polyclinics becoming the preferred referral centre for discharged patients [11]. Only 300 GPs work in public polyclinics and they would see an average of 58 patients per day compared to 30 per day seen by private GPs [29]. However, private GPs account for 80% of all of the GPs in Singapore, and so recruiting private GPs to the management of diabetic patients was considered an essential part of right-siting patients [11, personal communication]. In 2005, the SingHealth DOT Programme was established to integrate the private GPs into the traditional referral pathway. Over the next three years, it is projected that 140 GPs will be actively involved in the programme [17].

The SingHealth DOT programme aims to actively support the right-siting of clinically stable patients from the SGH Diabetes Centre to private DOT GPs to reduce unnecessary utilisation of expensive specialist resources, and to reduce waiting times for the large numbers of diabetic patients with complications who need medical care from hospital endocrinologists [personal communication]. It is a collaborative effort involving key stakeholders from the public and private healthcare sectors, professional bodies, such as the College of Family Physicians Singapore and Diabetic Society of Singapore (DSS), as well as the pharmaceutical industry [personal communication].

An important component of the DOT programme is the CDMO, established in April 2008. The CDMO drives the right-siting process through the SingHealth institutions by working closely with SingHealth’s IT team, developing a fully customised DOT IT system to replace the mostly manual tracking and follow-up system previously used by RSOs [personal communication]. The DOT IT system incorporates an e-referral system for GPs, it supports informed care, and the system facilitates receiving of regular updates from the GPs of right-sited patients [personal communication]. The Ministry of Health resourced the IT system and has begun to expand it in other regional networks becoming a universal DOT IT system [personal communication].

The two core components of the DOT Programme are the DOT Symposia and the DOT Optimisation Programme. The purpose of the DOT Symposia is to enhance GPs’ awareness of the benefits of the DOT programme by continuing medical education in key aspects of chronic disease management covered in four modular sessions: (i) lifestyle behaviour modification, (ii) healthy eating, (iii) medication and compliance and managing complications, and (iv) special needs in diabetes [personal communication]. Experts in community-based care for chronic diseases are invited to the Symposia to discuss and share their experiences with the participating GPs [personal communication]. The training sessions are sponsored by pharmaceutical companies, and 50% can be done via e-learning [personal communication].

The DOT Optimisation Programme supports GPs in managing people with diabetes, and is a partnership between SingHealth, Johnson & Johnson Medical Singapore and the DSS [personal communication]. It requires each participating GP to enrol three people with diabetes into the programme, and work towards achieving the DOT performance measures. The GPs have to sign up their enrolled patients for three customised education and counselling sessions by nurse educators from the DSS [11, 17, personal communication]. This diabetes education service sponsored by Johnson & Johnson covers diet, lifestyle modification, medication, and insulin therapy and skills [personal communication].

Specialists from the SGH Diabetes Centre refer clinically stable patients to RSOs located in the Diabetes Centre [11, personal communication]. The RSOs explain the DOT Programme to the referred patients. If the patient agrees to participate in the programme, RSOs will arrange for the patient to be followed-up by a private DOT GP [personal communication]. The DOT GP will develop a one-year patient management plan and send updates on the patient’s health to the referring specialist for shared care follow-up [16]. RSOs monitor right-sited patients and the DOT GPs for about a year [11, 17]. The clinical outcomes (glycated haemoglobin [HbA1c], blood pressure [BP], low-density lipoprotein-cholesterol [LDL-c]) of enrolled patients are tracked during this period [11] to ensure that DOT GPs provide good ambulatory care [11, 17, personal communication]. Family members of right-sited patients have been observed to seek medical care from DOT GPs [personal communication].

Due to the higher out-of-pocket expenditure incurred by patients in the care of private GPs, the CDMO developed three incentive initiatives to support patients choosing to participate in the programme: (i) Subsidised Drug Delivery Programme, (ii) Diagnostic Tests Incentive Programme, and (iii) Allied Healthcare Incentive Programme [11, 17, personal communication].

The Subsidised Drug Delivery Programme ensures that right-sited patients can continue to purchase subsidised drugs from the hospital even when under the care of private GPs [17, personal communication]. When DOT GPs fax the prescriptions of patients to the hospital’s pharmacy, the prescription drugs will be sent to the patients’ homes with only a courier charge of S$8 (£4) per trip [17].

The Diagnostic Tests Incentive Programme ensures that, once patients are discharged to DOT GPs, DOT GPs provide patients with a DSS referral letter for retinal and foot screening, and other optional services if required. DSS is a non-profit organisation that supports the DOT Programme by mobilising a bus for retinal and foot screenings. The bus travels twice yearly throughout Singapore to provide services near DOT GPs’ clinics, at the DSS’s Centres or the Changi General Hospital Community Health Centre [personal communication]. DSS sends the completed patient test results to the DOT GP and CDMO, and CDMO updates the test results into the DOT IT system [personal communication]. Right-sited patients do not have to pay for retinal and foot screening, but do have to pay for other optional services, such as lipid measurement and specialised care by a dietician [personal communication].

Finally, the Allied Healthcare Incentive Programme is a collaboration with Innovative Diagnostics Pte Ltd (IDPL) to provide the required laboratory tests (LDL, HbA1c and serum creatinine) according to the Ministry of Health’s components of care for good management of chronic diseases by DOT GPs [personal communication]. DOT patients are given blood test vouchers upon discharge, which are redeemed by the private DOT GP when blood tests are requested [personal communication]. IDPL sends the patient’s laboratory test results to the DOT GP and to the CDMO [personal communication]. CDMO pays IDPL for the required laboratory tests, but patients pay for additional tests requested. The right-sited patients benefit from reliable tests performed by IDPL, which cost less compared to usual charges by DOT GPs [personal communication]. DOT GPs benefit from the reduced administrative burden and regular visits by patients. The test results enable DOT GPs to provide the appropriate follow-up care when the patient next visits and CDMO receives timely data to develop patient-centric programmes [personal communication].

Since the introduction of the DOT programme, the average waiting time for a new appointment for subsidised patients at the SGH Diabetes Centre has been reduced from eight months in 2005 to 28 days [18]. Chronic disease patients now have easy access to a wider pool of well-trained GPs located near their neighbourhoods to help manage their condition, and benefit from shared care by GPs and specialists [16]. Processes are in place for patients to be referred back to the hospital by DOT GPs if necessary, and patients can continue to benefit from subsidised medical fees at the public hospital [18, personal communication].

The proportion of patients reaching optimal glycaemic control (HbA1c ≤7%) increased from 26% before enrolment to 51% after enrolment in the programme, and the increase was statistically significant [11]. The proportion of patients achieving optimal control for BP and LDL-c increased after enrolling in the programme, but did not reach statistical significance [11].

SingHealth conducted a study comprising two groups of people with diabetes: clinically stable patients discharged from a hospital specialist outpatient clinic (SOC) for follow-up by DOT GPs, and patients that could have been discharged but continued follow-up in the hospital SOC. In the group discharged and group remaining at the hospital, 81% and 79% of the patients maintained a clinically stable condition, respectively [personal communication]. When comparing the third visit to the second, the proportion of clinically stable patients was 97% in the discharged and 93% in the group remaining in the hospital [personal communication]. Notably, there were no statistically significant differences between the two groups in regard to the proportion of patients who were clinically stable at the second and third follow-up visits [personal communication]. This demonstrates that care provided by DOT GPs is comparable to care provided in the hospital for clinically stable patients [personal communication].

## Lessons from the SGH DOT Programme

In SingHealth, right-siting was enabled through a variety of patient incentives, such as laboratory test vouchers and subsidised drugs, easing the burden of anticipated out-of-pocket expenditure that would be usual under the care of private GPs [17, personal communication]. RSOs maintained a general oversight of the patient pathway and ensured appropriate and timely referral to private GPs. The integrated system ensured access to named specialists via phone, on-line or joint clinics [17] and supported shared care follow-up by enabling DOT GPs to share updates on the patients’ health status with the referring specialists [personal communication]. The Chronic Disease Management Programme allowed patients to use their Medisave, a compulsory savings programme, to pay for part of their outpatient chronic care management for diabetes, hypertension, hyperlipidaemia and strokes, promoting the involvement of private GPs in managing chronic diseases [11]. As a result, the shift of patient care from tertiary to primary care released resources for more complex cases to be dealt with and engaged a cadre of primary care professional that had operated in general outside of the public system.

The process, however, is not without its tensions. There is little incentive for public hospitals, with their fixed allocated budgets, to right-site their patients. A simulation study conducted at the SGH Diabetes Centre estimated that the revenue of the hospital would drop by S$1.9 million (£0.97 million) per year if simple cases were replaced with complex cases [13]. If simple cases were right-sited and time freed up was used by staff to conduct teaching and research, this would lead to an even greater fall in revenue by S$5.2 million (£2.64 million) per year [13]. This has been noted by the Health Minister who mentioned that there is a need for additional incentives to fund preventive care, integration and right-site patients [30].

Although SingHealth has delivered integrated diabetes care for years, there have not been any published studies that specifically assessed the effectiveness of integrated care in reducing costs and improving patient outcome and satisfaction. Potential cost savings can probably only be evaluated after a long period of providing integrated care as diabetes is a chronic disease, and the potential benefits of minimising complications, hospital admissions and utilisation of specialist care will be observed only in the long-term. SingHealth plans to analyse patient satisfaction for those enrolled in the DOT Programme in April 2012 [personal communication].

The type of integration that occurred with the SGH DOT Programme could be defined as functional, clinical, normative, horizontal, virtual and coordinated—improving ways of working across traditional health service boundaries. Functional integration is supported by the DOT IT system that enables sharing of patient information between the specialists, GPs and CDMO [master’s thesis]. Clinical integration is present as care by providers is integrated into a coherent process across professions with a clear programme structure, while normative integration is demonstrated by the shared vision among healthcare providers [master’s thesis]. Horizontal integration is observed as healthcare professionals that are usually from secondary care are brought together through agreements with the DSS and IDPL [master’s thesis]. Virtual integration is supported by the CDMO that coordinates care provided by different healthcare professionals, and the degree of integration is coordinated as there are RSOs to facilitate the transition of diabetes patients between care settings and there is regular exchange of information [master’s thesis]. This suggests that integration is a vague term used generically as a catch-all description for some health service innovations. Some attention to understanding what kind of integration is occurring is useful.

## Conclusion

Many developed countries are motivated to shift the care for people with diabetes from the hospital to the community, and to provide integrated and holistic care for patients. Developing a programme with a coherent process across all healthcare providers similar to the SGH DOT Programme may facilitate such efforts. The SGH DOT Programme facilitates the process of shifting clinically stable diabetic patients to DOT GPs, which enable patients to receive care closer to their homes and shorten the waiting time for patients with complications who need to see specialists in the hospital. It is important to recognise that successful integration may require incentives for institutional partners, as well as for the patients themselves.
